# Population prevalence of asthma and its determinants based on European Community Respiratory Health Survey in the United Arab Emirates

**DOI:** 10.1186/1471-2466-12-4

**Published:** 2012-02-16

**Authors:** Bassam H Mahboub, Suleiman Al-Hammadi, Mohamed Rafique, Nabil Sulaiman, Ruby Pawankar, Abdulla I Al Redha, Atul C Mehta

**Affiliations:** 1 Department of Pulmonary Medicine, Rashid Hospital, Dubai, United Arab Emirates; 2 Department of Pediatrics, Faculty of Medicine and Health Sciences, UAE University, P.O.Box 17666 Al-Ain, United Arab Emirates; 3 Department of Family & Community Medicine, Sharjah University, Sharjah, United Arab Emirates; 4 Allergy and Rhinology, Nippon Medical School, Tokyo, Japan; 5 Department of Otorhinolaryngology, Al-Baraha Hospital, Dubai, United Arab Emirates; 6 Chief Medical Officer, Shaikh Khalifa Medical City, Abu Dhabi, United Arab Emirates

## Abstract

**Background:**

No population study has explored the population distribution of adult asthma in the United Arab Emirates (UAE). The objective is to estimate asthma prevalence in general population in UAE.

**Methods:**

Using standard European Community Respiratory Health Survey (ECRHS) questionnaires and tools, this is a cross-sectional assessment of a random sample of the population in established quotas of the seven Emirates in the UAE. We surveyed 1,220 participants, of which 63.2% were male, and 20.1% were UAE Nationals, with a mean (SD) age of 32.9 (14.1) years.

**Results:**

Prevalence of individual respiratory symptoms from the ECRHS screening questionnaire in all participants were generally ranging 8 - 10%, while participants 20-44 years presented lower prevalence in all symptoms (*p *< 0.05). The expected male:female ratio of reported wheezing and asthma attacks and its treatment by age was not observed. Participating women reported more individual symptoms than men. Overall, there were 15.4% (95% C.I. 13.5 - 17.5) participants who fulfilled our screening criteria for asthma, while for consistency with ECRHS, there were 12.1% (95% C.I. 10.4 - 14.1) participants who fulfilled the ECRHS asthma definition, being 9.8% (95% C.I. 7.8 - 12.2) of those 20-44 years, that is 8.6% of male and 11.8% of female young adults participating.

**Conclusion:**

We conclude that asthma is common in the UAE, and gender differences are not observed in reported asthma symptoms in young adults. This being the first population based study exploring the prevalence of asthma and its determinants in the United Arab Emirates based on the ECRHS.

## Background

Asthma is a complex, chronic disease that can manifest at any age [1-3]. It has a very heterogeneous distribution, and its prevalence has been increasing in all regions of the World, likely due to a combination of factors, and many unknowns remain in determining asthma causation [4]. The disease represents a significant burden, not only in terms of morbidity and reduced quality of life of patients, but also in terms of healthcare cost [5,6].

Two international surveys of asthma prevalence have greatly enhanced our knowledge of the distribution and risk factors of asthma worldwide. First, the International Study of Asthma and Allergies in Childhood (ISAAC) [7], conducted in children of 6-7 and 13-14 years. Later, the European Community Respiratory Health Survey (ECRHS) [8], surveying that 20-44 years old, also led to an increased understanding of the worldwide distribution of asthma in young adults. Whilst Western Europe has some of the highest prevalence rates of asthma in the World, the ISAAC study found that the prevalence is not only high in English-speaking countries like the UK, Australia, New Zealand, and the US, but also identified new hot spots with higher than expected asthma in participating centers in other countries in Latin America, Asia, and elsewhere [9]. There is a scarcity of data about the distribution of asthma in the Gulf and near. Although studies have been carried out, they involved relatively small samples of patients selected from a limited number of clinical or other institutional settings, and latest data is not available. In UAE, in particular, there was an ISAAC asthma center, which reported a prevalence of physician-diagnosed asthma of 13% in 3,200 children aged 6-13 years in the seven Emirates of the UAE [10]. There have been other small studies surveying asthmatic children. However, to our knowledge no population study has explored the population distribution of adult asthma in the UAE. By using the screening questionnaire and tools of the ECRHS, we aimed to determine the prevalence of asthma symptoms in the general population in the UAE.

## Methods

This is a cross-sectional assessment of a random sample of the population of the UAE. The UAE lies within the Arabian Gulf Region, with seven Emirates, namely Abu Dhabi, Dubai, Sharjah, Ajman, Umm Al-Quwain, Ras Al-Khaimah and Fujairah.

In 2009, the UAE population was estimated at 6 million, of which fewer than 20% were UAE nationals or Emiratis [11]. The country's net migration rate stands at 22.98 per 1,000 inhabitants, the World's highest. The population of the UAE has a skewed sex distribution consisting of more than twice as many males as females, and in the 15-65 years age group it has a male:female ratio of 2.743. The UAE's gender imbalance is only surpassed by other Arab countries in the Gulf region.

The methodology and objectives of the ECRHS have been described previously [12]. Summarising, a random sample of the population aged 20-44 years is contacted and requested to complete a short screening questionnaire on respiratory symptoms. In a second phase of the study a 20% random sub-sample of the sample population and all remaining subjects reporting respiratory symptoms in the screening questionnaire and not yet included in the random sub-sample are asked to complete a long questionnaire and other tests.

### Population sampling and ethics

We included in the study male and female individuals of all ages in the seven emirates of the UAE. The sample size was calculated with a precision level of 3%, population size of 6 million, 95% Confidence Interval, and estimated asthma prevalence of 13%. Overall, 1,225 individuals were selected according to the following pre-determined sampling quotas by gender (66.5% males and 33.5% females); age (26.1% 0-19 year old, 72% 20-59 year old and 1.9% ≥ 60 year old); residency status (20.1% nationals and 79.8% expatriates);and geography within the UAE (34.1% Abu Dhabi, 32.2% Dubai, 19.3% Sharjah, 5% Ajman, 1.2% Umm Al-Quwain, 5.1% Ras Al-Khaimah, and 3.1% Fujairah). Given the absence of usable census information in the UAE, interviews were conducted in public locations across the seven emirates including town centers, malls, outside mosques, and industrial areas from late January 2010 to early March 2010. Apart from the inability to signing the informed consent, there were no exclusion criteria in this research. All participants were informed of the voluntary nature of this research and signed an informed written consent. This research protocol was approved by Clinical Research Ethics Authority of Dubai, UAE.

### Questionnaire

The English versions of the screening and long ECRHS questionnaires [12] were translated to Arabic, and participants were interviewed in a side-by-side bilingual version, to assess the presence of respiratory symptoms, other conditions, smoking, and treatments. As the interest was to use the ECRHS screening questionnaire in the most sensitive way as possible, any individual was considered symptomatic of asthma if he/she answered YES to any of the following questions: "Have you had wheezing or whistling in your chest at any time in the last 12 months?", or "Have you been coughing constantly for more than 3 weeks in any time in your life?", or "Have you had an attack of asthma in the last 12 months?", or "Do you have any nasal allergies including hay fever?". In addition any smoker or anyone answering YES to 50% of the eight questions of the ECRHS screening questionnaire was qualified for the survey.

All interviews were administered by an interviewer, and participants had the option to answer questions in the language they were more comfortable with, either Arabic or English. Results of the ECRHS screening questionnaire only are presented in this report.

### Statistical analysis

Data are presented as mean and standard deviation (SD) for continuous variables, or percentage for qualitative variables, as appropriate. Differences within groups were compared with analysis of variance (ANOVA) for continuous variables, and Chi^2 ^for categorical variables. The prevalence of asthma symptoms and their 95% confidence intervals (95% C.I.) was estimated for all participants, and by gender and age bands. Due to the sampling quotas by age, and also to compare directly with ECRHS centers, the most relevant results are also presented for participants 20-44 years, and younger and older ages. A *p *< 0.05 was considered statistically significant.

## Results

**Figure 1:**
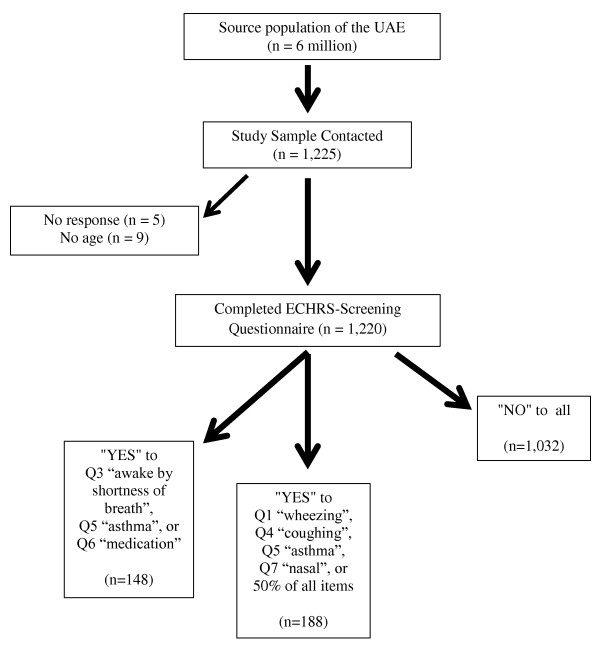
**CONSORT flow-chart of participants**.

The total number of respondents interviewed with sociodemographic data was 1,220 (Figure 1), of which 63.2% were male, and 20.1% were UAE Nationals, with a mean (SD) age of 32.9 (14.1) years, all according to the above mentioned quotas. In detail, sampling percentages of participants per Emirate was 34.1% Abu Dhabi, 32.2% Dubai, 19.3% Sharjah, 5.0% Ajman, 1.3% Umm Al-Quwain, 1% Ras Al-Khaimah, and 3.1% Fujairah.

Results by gender and age are presented for participants 20-44 years, younger and older ages (Table 1).

**Table 1:** Demographic characteristics of participants, by age band

Variable	19 yr or less n = 237 (19.4%)	20 to 44 yr n = 702 (57.5%)	45 yr or more n = 281 (23.0%)	ALL n = 1220
Male gender, n (%)	139 (58.6%)	440 (62.7%)	192 (68.3%)	771 (63.2%)

Age in years, mean (SD)	13.8 (4.9)	31.6 (7.0)	52.3 (5.5)	32.9 (14.1)

Prevalence of individual respiratory symptoms according to the ECRHS screening questionnaire in all participants were ranging from 8 to 10%, except for, lower for nasal allergies (6.8%) (Table 2). However, when stratified by age band, it can be seen that in participants younger than 20 years, as well as those older than 44 years, prevalence of nearly all symptoms are 10% or higher, while those participants 20-44 years presented lower prevalence in all symptoms (*p *< 0.05).

**Table 2:** Symptoms according to the ECRHS screening questionnaire in participants, by age band

**ECRHS screening questionnaire symptoms within the last 12 months**, n (%)	19 yr or less n = 237 (19.4%)	20 to 44 yr n = 702 (57.5%)	45 yr or more n = 281 (23.0%)	ALL n = 1220
Wheezing/whistling	31 (13.1%)	57 (8,1%)	34 (12.1%)	122 (10.0%)
Wheezing with breathlessness	27 (11.4%)	50 (7.1%)	32 (11.4%)	109 (8.9%)
Wheezing without a cold	24 (10.1%)	43 (6.1%)	31 (11.0%)	98 (8.0%)

Woken up with chest tightness	32 (13.5%)	53 (7.5%)	34 (12.1%)	119 (9.8%)

Woken up by shortness of breath	30 (12.7%)	51 (7.3%)	30 (10.7%)	111 (9.1%)

Woken up by an attack of coughing	30 (14.8%)	59 (8.4%)	33 (11.7%)	127 (10.4%)

Attack of asthma	28 (11.8%)	42 (6.0%)	27 (9.6%)	97 (8.0%)

Currently taking asthma medications	22 (9.3%)	46 (6.6%)	29 (10.3%)	97 (8.0%)

Nasal allergies (including hay fever)	22 (9.3%)	46 (6.6%)	15 (5.3%)	83 (6.8%)

**Figure 2:**
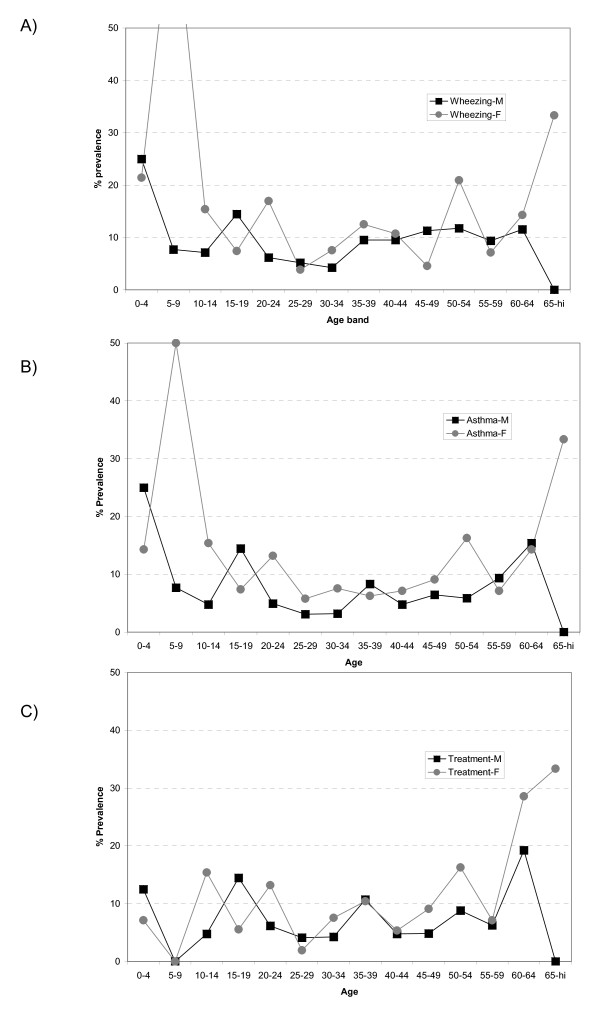
**Prevalence of wheezing (A), asthma attack (B) and asthma medication (C) by age, in male and female**.

We explored the male:female ratio of reported wheezing and of asthma attacks and its treatment by age (Figure 2). The classical inversion of the male:female ratio in adolescence was not observed; in girls 5-15 years wheezing and asthma attacks were more common than in boys, while prevalence of these symptoms were evenly reported up to the age of 60 years and older. Surprisingly, reported asthma treatment showed no gender ratio and was unchanged in all age bands, namely 9.3% in participants younger than 20 years, 6.6% in participants 20-44 years, and 10.3% in participants older than 44 years (Table 2).

These respiratory symptoms according to the ECRHS screening questionnaire are next presented in participants 20-44 year, by gender (Table 3). It can be seen that all symptoms were reported more frequently in female than male, differences being statistically significant only for "Woken up by an attack of coughing" and for "Nasal allergies (including hay fever)" (*p *< 0.05).

**Table 3:** Symptoms according to the ECRHS screening questionnaire in participants 20-44 yr, by gender

ECRHS screening questionnaire symptoms within the last 12 months, n (%)	20 to 44 yr male (n = 440)	20 to 44 yr female (n = 262)	*p*
Wheezing/whistling	30 (6.8%)	27 (10.3%)	0.069
Wheezing with breathlessness	26 (5.9%)	24 (9.2%)	0.253
Wheezzing without a cold	23 (5.2%)	20 (7.6%)	0.255

Woken up with chest tightness	28 (6.4%)	25 (9.5%)	0.080

Woken up by shortness of breath	31 (7.0%)	20 (7.6%)	0.440

Woken up by an attack of coughing	29 (6.6%)	30 (11.5%)	0.034*

Attack of asthma	21 (4.8%)	21 (8.0%)	0.058

Currently taking asthma medications	26 (5.9%)	20 (7.6%)	0.230

Nasal allergies (including hay fever)	21 (4.8%)	25 (9.5%)	0.011*

"*" Significant *p*-value

Overall, there were 184 (15.4%, 95% C.I. 13.5 - 17.5) participants who fulfilled our screening criteria for asthma (Figure 1). These asthmatics were more frequently male (56.5%) but there were no significant differences by age in male versus female asthmatics (Table 4). As per the results in the general population, there were neither clinical nor statistical differences in the distribution of individual respiratory symptoms by gender in these asthmatics, except for "Wheezing without a cold" (*p *< 0.05) (Table 4).

**Table 4:** Characteristics of those responding "YES" to Q1 "wheezing", Q4 "coughing", Q5 "asthma", or Q7 "nasal" according to the ECRHS screening questionnaire (n = 184)

	male (n = 104)	female (n = 80)	ALL n = 184	*p*
Age in years, mean (SD)	34.0 (15.4)	31.3 (15.7)	32.8 (15.5)	0.247

Age band, n (%)				0.309
• 19 yr or less	27 (26.0%)	18 (22.5%)	45 (24.5%)	
• 20 to 44 yr	47 (45.2%)	45 (56.3%)	92 (50.0%)	
• 45 yr or more	30 (28.8%)	17 (21.3%)	47 (25.5%)	

**ECRHS screening questionnaire symptoms within the last 12 months, n (%)**				

Wheezing/whistling	68 (65.4%)	57 (67.9%)	125 (66.5%)	0.758
Wheezing with breathlessness	60 (57.7%)	51 (60.7%)	111 (59.0%)	0.916
Wheezzing without a cold	59 (56.7%)	39 (46.4%)	98 (52.1%)	0.043*

Woken up with chest tightness	57 (54.8%)	44 (52.4%)	101 (53.7%)	0.770

Woken up by shortness of breath	59 (56.7%)	42 (50.0%)	101 (53.7%)	0.380

Woken up by an attack of coughing	70 (67.3%)	60 (71.4%)	130 (69.1%)	0.634

Attack of asthma	52 (50.0%)	47 (56.0%)	99 (52.7%)	0.464

Currently taking asthma medications	54 (51.9%)	41 (48.8%)	95 (50.5%)	0.769

Nasal allergies (including hay fever)	45 (43.3%)	40 (47.6%)	85 (45.2%)	0.559

"*" Significant *p*-value

For consistency with ECRHS sampling methodology, results in those participants responding YES to the following three questions: "Have you been woken by an attack of shortness of breath at any time in the last 12 months?", or "Have you had an attack of asthma in the last 12 months?", or "Are you currently taking any medicine (including inhalers, aerosols or tablets) for asthma?" are presented in Table 5. There were 146 (12.1%, 95% C.I 10.4 - 14.1) participants who fulfilled the ECRHS asthma definition in all ages. Specifically, the ECRHS asthma prevalence in those 702 participants with 20-44 years was 9.8% (95% C.I. 7.8 - 12.2), that is in males 8.6% (95% C.I. 6.2 - 11.7) and in females 11.8% (95% C.I. 8.2 - 16.4) participating.

**Table 5:** Characteristics of those responding "YES" to Q3 "shortness of breath", Q5 "asthma", or Q6 "treatment of asthma", according to the ECRHS main questionnaire (n = 146)

	male (n = 84)	female (n = 62)	ALL n = 146	*p*
Age in years, mean (SD)	30.8 (16.7)	33.9 (16.2)	32.6 (16.4)	0.263
Age band, n (%)				0.711
• 19 yr or less	22 (26.2%)	17 (24.4%)	39 (26.7%)	
• 20 to 44 yr	38 (45.2%)	31 (50.0%)	69 (47.3%)	
• 45 yr or more	24 (28.6%)	14 (22.6%)	38 (26.0%)	

**ECRHS screening questionnaire symptoms within the last 12 months, n (%)**				

Wheezing/whistling	59 (70.2%)	49 (76.6%)	108 (73.0%)	0.457
Wheezing with breathlessness	53 (63.1%)	45 (70.3%)	98 (66.2%)	0.649
Wheezzing without a cold	52 (61.9%)	34 (53.1%)	86 (58.1%)	0.037*

Woken up with chest tightness	56 (66.7%)	38 (59.4%)	94 (63.5%)	0.392

Woken up by shortness of breath	67 (79.8%)	44 (68.8%)	111 (75.0%)	0.131

Woken up by an attack of coughing	54 (64.3%)	44 (68.5%)	98 (66.2%)	0.603

Attack of asthma	52 (61.9%)	47 (73.4%)	99 (66.9%)	0.161

Currently taking asthma medications	56 (66.7%)	42 (65.6%)	98 (66.2%)	1.000

Nasal allergies (including hay fever)	29 (34.5%)	27 (42.2%)	56 (37.8%)	0.394

"*" Significant *p*-value

Compared to results in Table 4, these ECRHS-definition asthmatics were also more frequently male (57.5%), but again there were no significant differences by age in male versus female ECRHS-definition asthmatics. The distribution of individual respiratory symptoms by gender in these asthmatics were evenly distributed, again except for "Wheezing without a cold" (*p *< 0.05) (Table 5)

**Figure 3:**
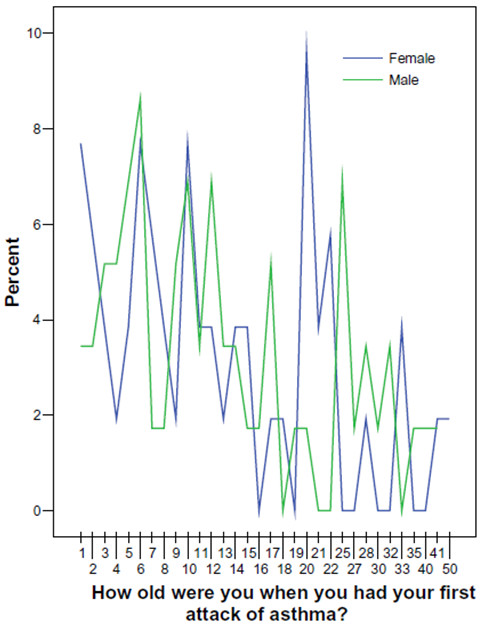
**Self-reported age at first attack of asthma in male and female asthmatics (n = 110)**.

Finally, an approximation to asthma incidence is presented in Figure 3, from birth to age 50 years. As expected, the reported age of the first attack of asthma occurred more frequently in childhood and adolescence, but in males there was a peak in asthma incidence (10%) after the age of 20 years, while in females there was another peak in asthma incidence (7%) after the age of 25 years.

## Discussion

We report in here the first population study assessing the distribution of adult asthma in the UAE. By using standard ECRHS questionnaires and tools, we determined that the prevalence of asthma (see Methods for definition) is at least 9.8% in young adults 20-44 years, and participating women reported more individual respiratory symptoms than men. As previously reviewed, only our national ISAAC center reported standard data on asthma in the UAE previously, but adult asthma information was yet unavailable. Recently, Alsowaidi et al. reported indirectly that in a randomly selected, age-stratified cohort of adolescent school children and their caretakers in the UAE, with a median age of 30 years (range 8 - 93 years), a 7.3% prevalence of comorbid allergic rhinitis asthma, lower in immigrants and with increasing age [13].

Our group recently participated in an international endeavor to explore the insights and realities of already diagnosed and treated asthmatics within the Gulf region [14], and results are also available for the UAE only [15]. Similar to asthmatics in other regions, it was concluded from surveying 200 asthmatics in the UAE that asthma burden and uncontrolled asthma were frequent, with 52.8% of the children and 17.1% of the adults missing school and work due to asthma in the past year, respectively. The percentage of Emirati asthmatics that had emergency room visits within the past year was 27.5%, and 4% were hospitalized. Only 5.5% used inhaled corticosteroids in the past year, and 47.5% were on short-acting β_2_-agonists. Contrary to our results in here on asthma symptoms, 85% of participating asthmatics were male.

Strengths of our research include an appropriate sample size for a prevalence study, surveying all seven Emirates, and the use of standard ECRHS questionnaires and methodology. However, some limitations are worth exploring. Given the reality of the UAE, with so many expatriates and immigrants, and the absence of appropriate census, it was decided to invite randomly and survey participants in public locations, rather than a door-to-door, random-digit dialing, or other sampling procedure.

Exploring the natural history of asthma in UAE is of interest, as we detected a substantial number of female asthmatics, and an absence of the expected male:female ratio of asthma incidence, to be reverted after adolescence. The gender differences in prevalence of wheeze and asthma attack did not reach statistical significance because the true differences appear to be quite small to be significantly detected based on our sample sizes of males and females. For example, using our sample sizes, the power to detect a difference in the prevalence of asthma attack between males and females that is of the order of 6% (corresponding to the observe differences in prevalence, see Table 4) is only 10% while the power to detect a difference in the prevalence of asthma attack between 20-44 years old males and females that is of the order of 3% (corresponding to the observed difference, see Table 3) is 31%. Very recently, it was reported pooling data of 48 ECRHS centers from 22 countries that there was no gender difference in asthma severity when comparing two repeat surveys completed in 1993 and 2002, but it was suggested that asthma severity might be less stable in women than in men [16]. Further research on the timing of these symptoms and perhaps a relationship with active and passive smoking patterns, and exposure to known asthma triggers and other local exposures, are worth considering.

We conclude that asthma in the study sample of the UAE population is frequent, 8.6% in male and 11.8% in female young adults 20-44 years., and that gender differences in asthma deserve further research.

## Competing interests

The authors declare that they have no competing interests.

## Authors' contributions

BHM have made substantial contributions to conception and design, acquisition of data, analysis and interpretation of data and drafting the manuscript and revising it critically for important intellectual content. SAH have made substantial contributions to analysis and interpretation of data, drafting the manuscript, critical revision for important intellectual content, and rewriting the manuscript according to the reviewers opinions. MR and AA have made substantial contributions to conception of the study, acquisition of data, and have given their final approval of the version to be published. NS have made substantial contributions to analysis and interpretation of data, and have given final approval of the version to be published. RP and ACM have made substantial contributions to conception and design, analysis and interpretation of data and drafting the manuscript, and have given final approval of the version to be published. All authors read and approved the final manuscript.

## Pre-publication history

The pre-publication history for this paper can be accessed here:

http://www.biomedcentral.com/1471-2466/12/4/prepub
